# Detectability of Crown-of-Thorns Starfish and Consequences for Culling or Removal

**DOI:** 10.3390/biology14101391

**Published:** 2025-10-11

**Authors:** Morgan S. Pratchett, Ciemon F. Caballes

**Affiliations:** College of Science and Engineering, James Cook University, Townsville, QLD 4811, Australia; caballesc@triton.uog.edu

**Keywords:** crown-of-thorns starfish (CoTS), coral reefs, disturbance, management, surveillance, sampling

## Abstract

**Simple Summary:**

Crown-of-thorns starfish are native to coral reefs throughout the Indo-Pacific and predominantly eat reef-building corals. At greatly elevated densities, during population irruptions (or outbreaks), the cumulative feeding activity of crown-of-thorns starfish can rapidly deplete the abundance of common prey corals across large areas of reef habitat. Therefore, a concerted effort is being made to suppress densities of crown-of-thorns starfish by culling or removing individual starfish and thereby minimizing ongoing coral loss. However, crown-of-thorns starfish may be extremely cryptic, thereby limiting the effectiveness of these control programs. This study contributes to the growing number of studies that measure the detectability of this organism and explore the various factors that influence levels of detectability. The key finding of this research is that at least 20% of starfish evade detection even during highly intensive surveys in small sample areas, and it is difficult to know exactly what proportion is never seen. These results have ramifications for the effectiveness of surveillance and management, whereby only a limited proportion of starfish will be amenable to culling or removal.

**Abstract:**

Population irruptions of crown-of-thorns starfish (CoTS; *Acanthaster* spp.) represent a perennial threat to Indo-Pacific coral reefs, often causing extensive coral loss and contributing to reef degradation. Therefore, extensive efforts are being made to contain population irruptions of CoTS either by culling or removing individual starfish across large reef areas. However, the efficacy and effectiveness of these management strategies are inherently constrained by limited detectability, even among adult starfish. This study adds to the limited quantitative data on the detectability of CoTS based on two independent intensive experimental studies conducted on Australia’s Great Barrier Reef. During depletive sampling (where all CoTS detected were removed prior to re-surveying the same area) over 3 days at Lizard Island, a total of 96 (out of 132) CoTS were recorded during initial surveys, and the average detectability across 34 transects where CoTS were recorded was estimated to be 78.4% (±13.4 SE). Estimated detectability declined to 64.4% (±11.22 SE) on day 2, suggesting that the remaining CoTS were more cryptic. During mark–recapture studies at Rib Reef, the total sample population was estimated to comprise 411 individuals, of which 266 (64.7%) were sighted during initial (day-time) surveys, while 322 (78.3%) were sighted at night. Average detectability across all surveys was estimated to be 75.9% (±0.05 SE). Our findings reaffirm that the detectability of adult CoTS is limited, which will inherently constrain the effectiveness of culling and removal.

## 1. Introduction

Coral reef ecosystems are threatened by escalating disturbances and anthropogenic pressures, especially climate change [1,2]. Climate change represents an existential threat to coral reefs, with the most immediate effects being attributable to the increasing incidence and severity of marine heatwaves [3] that cause coral mortality and changes in coral assemblages at unprecedented (near global) scales [4,5,6,7]. Reducing global carbon emissions is therefore critical to conserve coral reef ecosystems [8]. However, the threat of climate change also provides a renewed imperative to strengthen localized and direct management actions [1,3], such as increasing the extent and effectiveness of marine protected areas, achieving water quality improvements, and suppressing populations of pest species. Localized and direct management interventions that minimize coral loss are particularly critical to maximize opportunities for natural adaptation of coral assemblages exposed to changing environmental conditions [9,10,11]. Population irruptions of crown-of-thorns starfish (CoTS; *Acanthaster* spp.) are one of the major causes of coral mortality throughout the Indo west-Pacific that are amenable to direct management [10,11].

**Figure 1:**
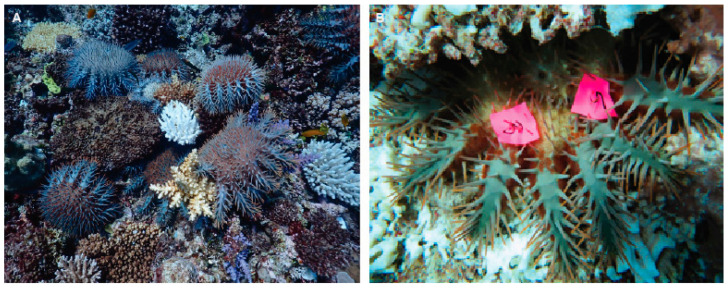
Population irruption of Pacific crown-of-thorns starfish (*Acanthaster* cf. *solaris*) on Australia’s Great Barrier Reef, with recent feeding activity apparent based on the white corals in the foreground (**A**). Crown-of-thorns starfish showing temporary tags used to mark individuals in mark–recapture surveys (**B**). Photographs by M.S. Pratchett.

*Acanthaster* spp. are native to coral reefs throughout the Indo-Pacific, though there are at least five recognized species with distinct geographic ranges [12,13]. The most extensively studied species, *Acanthaster* cf. *solaris* [13], for which further work is still required to confirm the appropriate nomenclature [12], is found on coral reefs in the Western Pacific, normally at low densities (≤3 starfish·ha^−1^; [14]). However, major population irruptions of *A.* cf. *solaris* have been recorded throughout their range, and the cumulative effects of high densities of adult crown-of-thorns starfish often leads to extensive coral depletion [15,16,17] (Figure 1). It has been estimated that densities > 15 CoTS·ha^−1^ cannot be sustained and will lead to net coral loss [18], though this will depend on coral cover and composition [19].

Recurrent population irruptions of *A.* cf. *solaris* represent a significant and persistent threat to coral assemblages on Australia’s Great Barrier Reef (GBR) [20]. Notably, there have been four distinct episodes of population irruptions since the 1960s [21,22], with renewed outbreaks of CoTS detected in 2021 [14,23]. Throughout this period there have been widespread declines in coral cover, with up to 42% of coral loss being attributed to population irruptions of CoTS [24,25]. Suppressing population irruptions of CoTS is considered the foremost opportunity to redress coral loss on the GBR [11], with models suggesting that coral cover would have increased until 2012 were it not for population irruptions of CoTS [24]. Accordingly, there has been significant and increasing investment in the GBR CoTS culling program, with the goal of reducing coral loss and enhancing reef resilience [11].

Effective and enduring management of CoTS requires deeper, more holistic consideration of the multitude of factors that contribute to population irruptions [26], though manual culling or removal of individual CoTS is the most direct and demonstrated method for suppressing CoTS densities [27,28,29]. The efficacy and effectiveness of CoTS culling and removal is, however, subject to many factors, including the biology and behavior of individual CoTS that effects detectability, which may vary at large spatial and temporal scales [30]. For the most part, CoTS shelter beneath corals or well within the reef matrix [31,32], emerging mainly to search for prey and feed on select coral colonies [33]. Feeding activity mainly occurs at night, especially for smaller CoTS [32], which constrains the effectiveness of culling activities conducted during daylight hours.

The purpose of this study was to assess the detectability of CoTS and their corresponding amenability to culling and removal using two different methods to estimate detectability. This study builds upon existing estimates of detectability [30,34,35] while furthering understanding of the factors that affect the detectability of CoTS, which is critical for managing population irruptions, especially if detectability varies spatially and temporally [36]. The first study involved depletive sampling of CoTS along fixed transects, with successive surveys conducted after 1–2 days to detect any CoTS that were ostensibly missed during subsequent surveys. The second experiment used mark–recapture protocols, whereby individual CoTS were marked with temporary tags that minimized interference to the animals. The local sample population of CoTS was then inferred based on the proportion of marked individuals that were re-sighted during subsequent surveys conducted within 48 h. Detectability in the latter study was estimated based on the number of CoTS sighted during the day and at night relative to the estimated sample population. Accounting for the limited detectability of CoTS is important for standardizing estimates of population density obtained using different sampling methods [34], especially where ecological models and management thresholds are based on absolute densities. However, existing estimates of CoTS detectability vary greatly, ranging from 22.7% to 88.2% [30,34,35]. Variations in detectability are partly attributable to differences among sampling methods [34] but may also be due to intrinsic (e.g., size-based differences in the behavior of CoTS) and extrinsic (local coral cover and habitat complexity) factors [30,35].

## 2. Materials and Methods

**Figure 2:**
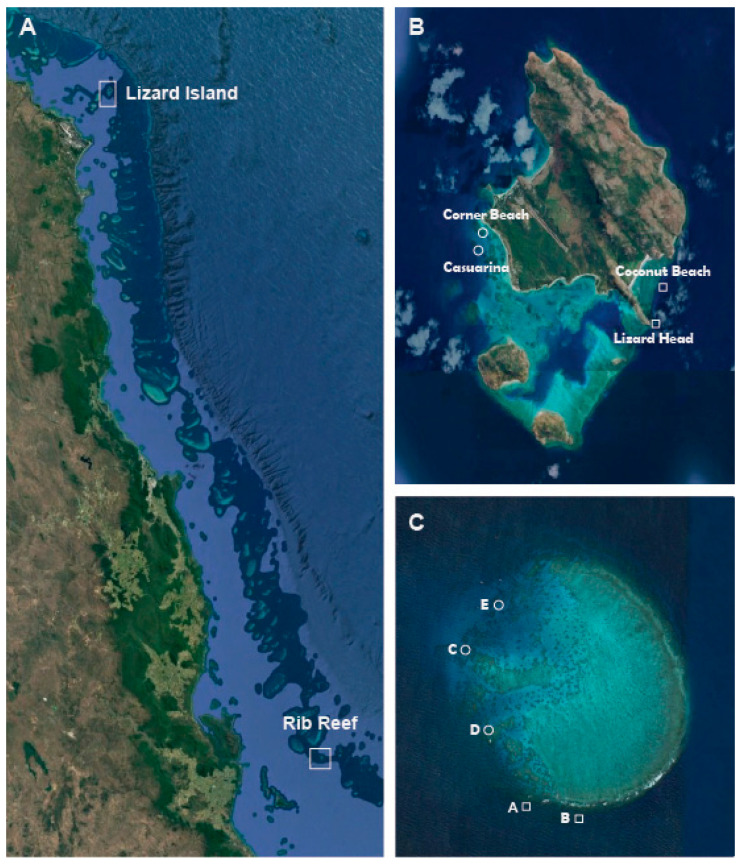
Satellite photographs from © Google Earth showing the position of the two distinct study reefs on the Great Barrier Reef (**A**), as well as specific sites at Lizard Island (**B**) and Rib Reef (**C**). Sites on the seaward (exposed) side of the reef are indicated with squares, while sites on the leeward (sheltered) side are indicated with circles.

This study reports on two independent experimental studies conducted at two dis-tinct locations and at different times on Australia’s Great Barrier Reef: Lizard Island (14°40′ S, 145°27′ E) in 1997 and Rib Reef (18°29′ S 146°52′ E) in the 2016–2017 period (Figure 2). While the experimental work was conducted up to 30 years prior to this study, the results are still relevant, especially given the limited data on the detectability of CoTS and contemporary considerations of management effectiveness. For both experiments, sampling was conducted along replicate 50 m belt transects that were marked throughout the limited course sampling (up to 5 days) by affixing fiberglass tapes to the reef substrate. Intensive searching was then undertaken up to 5 m on either side of the transect line to locate individual CoTS. All sampling was conducted on SCUBA, and two divers moved slowly along either side of the transect line (2 m·min^−1^), looking into crevices and overhangs from multiple angles to maximize the detection of CoTS. The lead author (MSP) was the lead diver for all surveys across both studies and ensured consistent sampling efforts across successive surveys. At both reefs, sampling was conducted across a range of sites with differing aspects (on the windward versus leeward sides of the reef) and in different reef zones, which were distinguished by the angle of the reef slope and depth (Figure 2).

### 2.1. Depletive Sampling at Lizard Island

The first experiment was conducted in 1997, following marked increases in local densities of CoTS at Lizard Island [36]. Sampling was conducted across four sites, which varied in reef aspect and corresponding exposure to prevailing winds and waves. Two sites (Lizard Head and Coconut Beach) were located on the south-east (windward) side, and two sites (Corner Beach and Casuarina) were located on the western (leeward) side of Lizard Island (Figure 2B). At each site, 5 replicate 50 m × 4 m transects (200 m^2^) were deployed on the shallow reef edge (0–3 m depth) and on the deeper and more steeply inclined section of the reef (4–9 m depth). To maximize detection of CoTS and test for observer bias, the entire area of each transect was surveyed by each of the two independent divers in quick succession. Successive sampling and removal of all CoTS were then conducted three times over 4–5 days. The objective of this study was to assess the precision and repeatability of survey methods, though it quickly became apparent that the detectability and amenability of CoTS to removal varied between days. All CoTS that were detected during each of three distinct surveys were collected and taken to the Lizard Island Research Station for use in tank experiments [37] before being disposed of on land.

### 2.2. Mark–Recapture Sampling at Rib Reef

Sampling at Rib Reef was conducted at 5 different sites over three distinct sampling periods (in December 2016, February 2017, and May 2017), when active outbreaks of *A.* cf. *solaris* were concentrated on mid-shelf reefs in the area between Cairns and Townsville [38]. Sampling was conducted at different sites during each sampling period to take advantage of prevailing conditions and maximize sampling across sites with different exposure conditions or aspects. At each site, sampling was conducted on three separate occasions along each transect; initial sampling was conducted between 0700 and 1700 h, establishing multiple transects that were then re-surveyed after dark (1830–2000 h). Subsequent surveys were then also conducted within 1–4 days, but only during daylight hours. The primary objective of this study was to assess fine-scale movement of individually tagged CoTS [39].

Overall, six transects were established within the reef crest habitat (2–3 m depth) and six within the reef slope habitat (6–8 m depth). CoTS were surveyed within 2.5 m on either side of the transects on the reef crest, where there was high cover of tabular *Acropora* spp. [31]. In deeper, less complex habitats, surveys were extended to 5 m on either side of the transect line (500 m^2^). Coral cover and composition were recorded by identifying hard corals (to genus) that were intersected by uniformly spaced points (0.5 m apart) along the length of each transect, giving 100 sampling points per transect. All CoTS that were found within the sample area were temporally tagged (Figure 1B) by piercing small (2 cm) pieces of flagging tape over the spines on accessible arms using 30 cm long forceps. The initial estimate of tag retention (from the first sampling site) where all CoTS (*n* = 75) were tagged with two independent tags was 94.1% [40].

### 2.3. Estimating and Comparing Detectability of CoTS

Detectability of CoTS was quantified based on the proportion of the total sample population (*N*) that was sighted or captured (*C*) during each independent survey. For depletive sampling at Lizard Island, the total sample population was estimated based on the cumulative number of CoTS that were removed across all successive surveys. This assumes that all CoTS initially present within the transect area were detected and removed during successive surveys conducted over 3–5 days. There may, however, be a small number of CoTS that evaded detection across all three intensive searches, which would lead to underestimates of the total sample population and corresponding overestimates of detectability. Conversely, there is a possibility that additional CoTS may have moved into the sample areas over the limited course of the study [33], which would also affect estimates of total sample population and detectability.

For the Rib Reef experiment, the sample population (*N*) on each transect was estimated independently for initial sampling conducted during the day and the subsequent nocturnal survey, using the Lincoln–Petersen mark-recapture method (*MC*/*R*), where the number of CoTS sighted and marked (*M*) is multiplied by the number of animals sighted in the subsequent survey (*C*) and divided by the number of marked individuals in that survey, effectively representing recaptures (*R*). Detectability during any given survey (e.g., initial surveys conducted during the day and at night) was then estimated based on the number of CoTS sighted relative to estimates of overall sample population on each transect. Independent estimates of the overall sample population (*N*) were obtained for each successive set of surveys, but the maximum *N* value was used to infer the total sample population. This assumes that there is no movement of CoTS in or out of the transect area and that all CoTS have an approximately equal chance of being sighted during each survey. Critically, however, this method explicitly accounts for the fact that some starfish may evade detection throughout the successive sampling process. 

Detectability of CoTS was analyzed using Generalized Linear Mixed Models (GLMM) with binomial distributions, whereby the number of CoTS that were sighted was directly compared to the total sample population (based on cumulative removals or MC/R estimates) on each transect. Alternative models were constructed with glmmTMB version 1.1.11 [41] in R 4.4.2 [42] (R Core Team, Vienna, Austria). The relevant factors that could be tested in each experiment (Table 1) were considered independently and collectively. All models include site as a random effect. Models of increasing complexity were compared using the Akaike Information Criterion corrected for small sample sizes (AICc) to the best fitting combination of categorical and continuous predictors using the MuMIn package version 1.48.11 [43]. Each model was validated, and goodness of fit was checked visually and statistically, while model convergence, collinearity and dispersion were checked using the performance package version 0.13.0 [44].

It was not possible to account for variation in the size of individual CoTS in our analyses of detectability because detectability was calculated based on the proportion of CoTS recorded at the scale of individual transects. There was also limited variation in the size structure of CoTS among sites or zones (Appendix A: Table A1 and Table A2). However, given that detectability is expected to vary with body size [36], we explicitly calculated the estimated sample population of very small (≤20 cm diameter), small (21–30 cm diameter), medium (31–40 cm diameter), and large (>40 cm diameter) starfish using *MC*/*R*, by pooling data across all transects from Rib Reef. The total recorded number of CoTS in each of the aforementioned size classes during the day and at night was then compared with the estimated sample population (*N*) to assess size-based variations in detectability.

## 3. Results

### 3.1. Depletive Sampling at Lizard Island

A total of 132 CoTS were removed during successive removals conducted on 40 replicate 50 m × 4 m transects at Lizard Island (in 1997), corresponding with a mean density of 3.30 CoTS per 200 m^2^ (±0.39 SE). Recorded densities of CoTS varied markedly among sites and zones, ranging from 0.2 CoTS per 200 m^2^ (±0.20 SE) on the reef crest at Coconut Beach to 7.2 CoTS per 200 m^2^ (±0.66 SE) on the reef slope at Casuarina, which is equivalent to 360 CoTS·ha^−1^ (Table A1). The diameter of CoTS averaged 34.47 cm (±0.74 SE), ranging from 19 to 51 cm, and was generally similar among sites and zones (Table A1).

**Figure 3:**
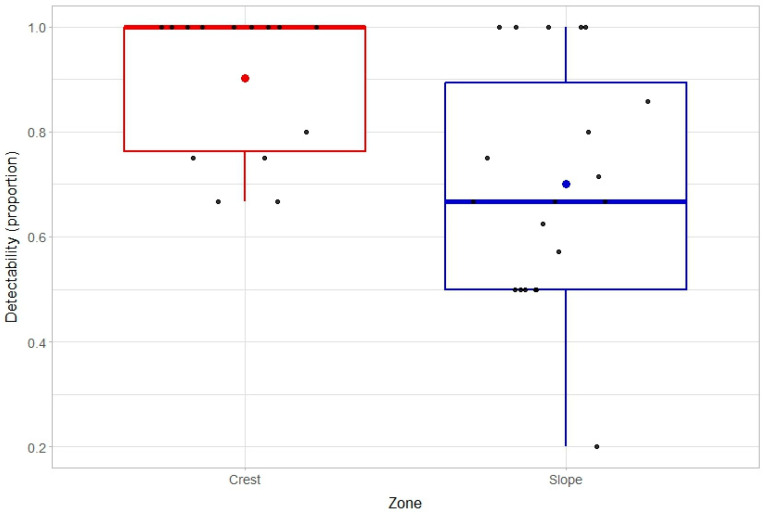
Detectability of Western Pacific CoTS (*A*. cf. *solaris*) on the reef crest (2–3 m depth) versus reef slope (6–8 m depth) during depletive sampling at Lizard Island. Variation in detectability between zones is displayed using Tukey’s box plot, where the thick horizontal line is the median and the box indicates the interquartile range. Large colored dots also show the mean, while smaller black dots indicate independent estimates for replicate transect.

The proportion of CoTS that were sighted and removed during initial surveys across all transects (*n* = 40) was 0.73 (96 out of 132 CoTS), which declined to 0.69 (25 out of 36 CoTS) for the second survey. Average detectability across transects and surveys where there was at least one CoTS presumed to be present (*n* = 34) was 0.74 (±0.04 SE), ranging from 0.00 to 1.00. Lowest levels of detectability (0.00) were recorded on transects where only a single starfish was presumed to present, and only after initial removals had been conducted. The resolution for estimates of detectability on transects with very low densities of CoTS is, however, inherently constrained (e.g., values can only be 0.0 or 1.0, where there is only a single animal present). Average detectability for just the initial survey was 0.78 (±0.13 SE), ranging from 0.20 to 1.00 (Figure 3).

The best model to account for the detectability of CoTS based on depletive sampling at Lizard Island included zone (glmmTMB(cbind(Find, Miss)~Zone + (1|Siteno)), family = binomial), with limited additional information provided when considering aspect (windward versus leeward sites), survey (first versus second survey), or cumulative number CoTS (on individual transects). Detectability during initial surveys was significantly different among zones (estimated fixed effect = −1.22, se = 0.53, *p* = 0.02), being higher on the reef crest (0.90 ± 0.04 SE) compared to the slope (0.70 ± 0.05 SE). It was also apparent that the densities of CoTS were consistently lower on the crest (1.1 CoTS per 200 m^2^ ± 0.24 SE) versus slope (3.1 CoTS per 200 m^2^ ± 0.38 SE).

### 3.2. Mark–Recapture Sampling at Rib Reef

A total of 357 CoTS were recorded across all surveys conducted on 12 replicate transects at Rib Reef. The combined areal extent of all transects (which varied from 250 to 500 m^2^) was 4500 m^2^, and the overall mean density (based on the cumulative number of CoTS recorded) was 954.24 CoTS·ha^−1^ (±0.39 SE). The maximum sample population (using *MC*/*R*) was estimated to be 411 CoTS (913.33 CoTS·ha^−1^) across all transects. The diameter of CoTS averaged 30.75 cm (±0.39 SE), ranging from 10 to 56 cm, and was generally similar among sites and zones (Table A2).

**Figure 4:**
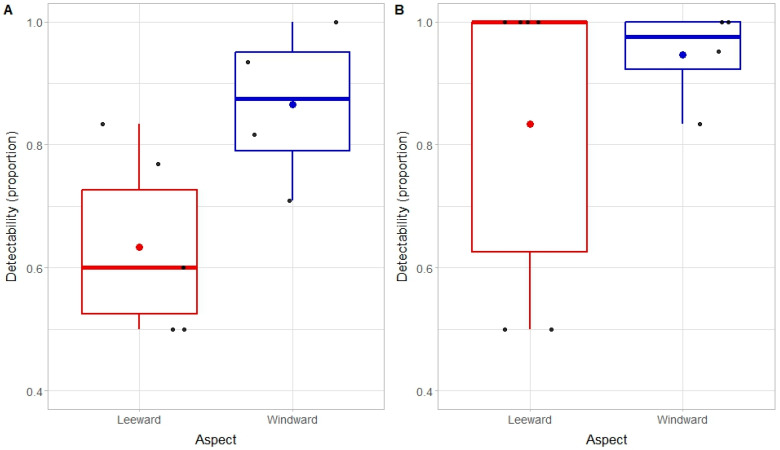
Detectability of Western Pacific CoTS (*A*. cf. *solaris*) during surveys conducted during the day (**A**) and at night (**B**) for sites located on the leeward versus windward sides of Rib Reef. Variation in detectability between sites with different aspects is displayed using Tukey’s box plot, where the thick horizontal line is the median and the box indicates the interquartile range. Large colored dots also show the mean, while smaller black dots indicate independent estimates for replicate transect.

The proportion of CoTS that were sighted (and tagged) during initial surveys conducted during the day (266 out of 411 CoTS) averaged 0.69 (±0.06 SE) across all transects where there was at least one starfish presumed to be present (*n* = 11). The proportion of CoTS detected during nighttime surveys on the same transects was 0.78 (322 out of 411 CoTS), averaging 0.76 (±0.10 SE) among transects. Average detectability across all transects and surveys where there was at least one CoTS presumed to be present (*n* = 22) was 0.76 (±0.13 SE), ranging from 0.50 to 1.00 (Figure 4).

The best model to account for detectability of CoTS based on mark–recapture methods at Rib Reef included time of day and aspect (glmmTMB(cbind(Find, Miss))~Time × Aspect + (1|Siteno), family = binomial), with limited additional information provided when considering zone (reef crest versus reef slope) or coral cover (on individual transects). There was a significant interaction between time and aspect (Type II Wald χ^2^ = 9.10, df = 1, *p* < 0.01), though detectability of CoTS was consistently higher at windward versus leeward sites (Figure 4). The increased detectability recorded at night versus during the day was, however, more pronounced at leeward sites.

While it was not possible to explicitly account for the size of CoTS in our analyses of detectability, the detectability of CoTS (estimated based on *MC*/*R*) consistently increased among size classes (Table 2). Detectability was also generally higher at night versus the day, except among very small size classes. Notably, the number of very small starfish (≤20 cm diameter) recorded at night was nearly twice that recorded during the day, and detectability remained very low.

## 4. Discussion

Recorded densities of CoTS at both Lizard Island and Rib Reef were well above indicative thresholds for population irruptions [18]. Moreover, actual densities are likely to be substantially higher given inherent constraints in detectability, whereby the maximum inferred density based on the estimated sample population was 3560 CoTS·ha^−1^ on individual transects at Rib Reef (Table A2). The detectability of CoTS clearly varies with the survey method [34], especially the scale of sampling. Estimates of detectability recorded herein were very similar for Rib Reef (0.76 ± 0.13 SE) and Lizard Island (0.78 ± 0.13 SE) despite using different methods to establish the sample population. This suggests that potential biases in estimates of abundance and detectability have limited or consistent effects. Indeed, one of the key assumptions of the current methods is that populations are essentially closed at the scale of independent sampling units (200–500 m^2^), and average displacement of individually tagged CoTS at Rib Reef was just 0.96 m, ranging from 0 to 22.8 m [40]. Our estimates of detectability were also comparable to that of other studies that used intensive searching on SCUBA within relatively small (200–250 m^2^) prescribed sampling areas (Table 3). Estimates of detectability using larger sampling units are much more constrained [30], though it is not clear whether this is solely attributable to changes in search intensity and efficiency as the methods used to infer the sample population also differ (Table 3).

Overall detectability of CoTS at Rib Reef was estimated to be 75.9%, suggesting that the true sample population was 25% higher than the number of CoTS recorded. Even more critically, the detectability on individual transects ranged from 0.20 to 1.00 across both studies, and this variability was largely independent of local CoTS densities. Limited detectability will inevitably constrain the capacity to comprehensively cull or remove CoTS during single or intensive management activities [47]. Critically, CoTS tend to move and feed mainly at night [31,32] and otherwise tend to shelter within the reef matrix. Recent research has also shown that CoTS may go 2–3 days without feeding or moving [48], remaining hidden throughout this period. Detectability of CoTS is therefore maximized when carefully searching inside the interstices of reef habitats [31,34] rather than simply recording starfish that are sufficiently exposed and amenable to rapid or “fly-over” surveys [30]. Even so, there may be some starfish that evade detection, sheltering out of site within very deep or complex holes in the reef matrix for several days, and they are therefore not amenable to culling or removal. Contrary to previous studies showing that cryptic behavior of CoTS was higher in shallow reef environments [31,45], we found that detectability at Lizard Island was higher on the reef crest compared to the reef slope. Detectability of CoTS is not necessarily directly related to the proportion of individuals that are exposed versus cryptic but is further influenced by the capacity to find cryptic individuals. The sheltering behavior of CoTS is seemingly motivated by the inherent avoidance of areas with high light intensity [49,50]. In shallow habitats with higher light intensity, it is possible that CoTS must move deeper into the reef matrix to seek sheltering sites with reduced light intensity and will therefore be much more difficult to detect using visual surveys.

Establishing what proportion of CoTS can be surveyed, culled, and/or removed requires an independent measure of the true sample population (*N*), which is often based on intensive and repeated sampling within prescribed areas [35,47]. However, detectability of CoTS is limited using even the most effective and intensive survey methods (Table 2), and corresponding estimates of detectability fail to account for the proportion of CoTS that evade detection throughout intensive or repeated visual surveys. Mark–recapture methods provide some insight into the proportion of individuals that evade detection during successive surveys [35] but assume that every individual has equal probability of being captured in any given sample [51]. The periodicity of sampling, if not the total duration of such studies, should therefore account for the period of time that CoTS remain hidden between feeding bouts or the changes in their position and exposure. While adult CoTS tend to exhibit regular diurnal patterns of feeding and exposure, leading to increased detection during crepuscular periods or at night [31,32,47], more research is needed to understand how often CoTS defer feeding for multiple consecutive days and why [48]. This is particularly critical for culling programs that attempt to maximize the proportion of CoTS that are culled or removed. Novel sampling methods, such as eDNA [23,52] and acoustic tracking [40], may provide further opportunities to assess the proportion of CoTS that evade detection even after multiple intensive searches (and removals) from given reef areas and further verify assumptions pertaining to current detectability estimates.

Inherent constraints in the detectability of CoTS pose an issue for culling and removal, though it may be possible to account for consistent biases in detectability associated with specific methods to standardize estimates of absolute density [36,53,54]. It is very challenging, however, to account for the varying detectability of CoTS [34], unless the factors that affect detectability are clearly defined and readily measurable. It is often expected that detectability of CoTS will vary with the structure of coral reef habitats, but studies explicitly testing for such relationships [30,35] have shown limited effects of coral cover or habitat complexity (Table 3). There has also been limited evidence that detectability varies with CoTS densities. Accordingly, variation in CoTS densities and coral cover did not explain the variation in detectability of CoTS among transects at Lizard Island and Rib Reef, respectively. This suggests that the capability of CoTS to seek shelter is not constrained by habitat structure, though it is possible that CoTS simply avoid reef habitats devoid of necessary sheltering opportunities.

Detectability of CoTS is largely affected by intrinsic variation in behavior, and in particular, size-based differences in propensity to feed and corresponding emergence from cryptic sheltering locations [29,30,31,32]. At Rib Reef, detectability of very small CoTS (≤20 cm) was ≤0.5 compared to >0.90 for larger CoTS, and though many more smaller CoTS were recorded during nocturnal surveys compared to diurnal surveys, detectability was still highly constrained. This corresponds very closely to asymptotic relationships reported in MacNeil et al. [35] and is partially explained by overall increases in exposure with increasing body size [30]. However, Pratchett et al. [30] showed that relationships between size and exposure varied regionally, and variation in the body size of CoTS does not explain why detectability was higher at windward versus leeward sites at Rib Reef. The opportunity to conduct intensive sampling at windward sites was, however, conditional upon persistent low winds, and which may account for observed differences in detectability, whereby CoTS are more likely to feed during calm conditions, especially at shallow windward sites.

### Future Research Directions

Acknowledging inherent biases and constraints of different survey methods, and corresponding uncertainty in estimating absolute abundance of CoTS, is critically important for advancing understanding and management of population irruptions. This is not to suggest that survey methods with limited capacity to accurately discern the absolute abundance of CoTS should not be used. Rather, different survey methods vary in their application to discern subtle changes in the abundance of CoTS that are indicative of the early initiation of population irruptions [14] versus large-scale patterns in the incidence of population irruptions [55]. However, data obtained using different survey methods and at different spatial scales is not necessarily comparable, and more work is needed to assess relevant biases of specific survey methods, including effects of changes in the size of sampling areas and the corresponding capacity to intensively search the entire sample area. Critically, effectively suppressing elevated densities of CoTS requires intensive and extensive culling or removal [11,29,56]. On the GBR, for example, CoTS culling activities are undertaken within non-overlapping polygons that average 14 ha in size [27]. Given apparent declines in detectability of CoTS when increasing the spatial extent of sampling units, it cannot be assumed that levels of detectability achieved by large-scale culling operations are equivalent to that reported in this study.

Research on the biology and behavior of CoTS is almost invariably undertaken during population irruptions [57], reflecting the importance of these phenomena in structuring coral reef ecosystems and contributing to coral reef degradation. When highly abundant, however, most researchers rely on instantaneous observations obtained from many distinct animals to characterize their biology and behavior [30,31,32]. However, intensive studies of individual CoTS provide different insights, revealing marked individualistic differences [33,48] that may be fundamental in understanding the initiation and collapse of population irruptions [42]. Improved understanding of changes in the behavior of CoTS (e.g., with increasing densities or reduced food availability) may also be used to improve the accuracy of surveillance and effectiveness of management activities.

## 5. Conclusions

This study reaffirms that it is very challenging to effectively survey crown-of-thorns starfish (CoTS) and obtain accurate estimates of absolute density [34,35,47], especially where populations comprise a wide range of different size classes. Even during intensive sampling within relative finite reef areas (up to 500 m^2^), a significant proportion of CoTS (>25%) may evade detection, including >50% of very small starfish (<25 cm diameter). It is also very difficult to account for CoTS that remain hidden for extended periods and will therefore not be amenable to surveillance or culling. Moreover, detectability is likely to be even more constrained when operating at larger spatial scales, as generally required for effective management [27]. It is therefore essential that sampling and monitoring programs are designed to inform and assess the effectiveness of ongoing CoTS management, using survey methods that provide necessary levels of detection and resolution. At the very least, much more consideration needs to be given to measuring detectability in the course of routine monitoring for all marine organisms [51].

## Figures and Tables

**Table 1:** Factors expected to affect the detectability of CoTS.

Factor	Potential Mechanism (Directionality of Effect)
Aspect ^L,R^	(−) CoTS are less likely to be exposed when subject to higher hydrodynamic forces and more likely to become dislodged [45,46]
Depth/zone ^L,R^	(+) Diurnal shifts in behavioral modality are less pronounced in low-light environments [45]
CoTS density ^L^	(+) Increased competition for food leads to increased feeding during the day [46]
Body size ^R^	(+) Larger CoTS feed more often and are more likely to be exposed, especially during the day [30,31,32,33,45]
Coral cover ^R^	(−) There are greater opportunities to remain hidden in areas with high coral cover [15]
Prey availability	(−) CoTS spend less time moving in search of prey in areas with high cover of preferred coral prey, especially *Acropora* [33]
Individual condition	(+) CoTS in poor condition spend more time searching for food [45]
Habitat complexity	(−) Highly complex habitats provide greater opportunities for CoTS to remain hidden [14,30]
Night versus day ^R^	(+) CoTS are more exposed when feeding, which tends to occur mostly at night [31,32,33]
Summer versus winter	(+) Temperature-mediated changes in metabolic demands increase time spent feeding [32]

^L^ Factors considered at Lizard Island; ^R^ factors considered at Rib Reef.

**Table 2:** Number of CoTS sighted and tagged (M) versus the estimated sample population (*N*) within each of the 4 distinct size classes during surveys conducted during the day and at night.

Size (Diameter)	Day			Night		
	M	*N*	Detectability	M	*N*	Detectability
Very small (≤20 cm)	9	18	0.50	16	36	0.44
Small (21–30 cm)	140	188	0.74	179	203	0.88
Medium (31–40 cm)	74	98	0.89	95	105	0.90
Large (>40 cm)	26	33	0.91	32	34	0.94

**Table 3:** Contrasting estimates of detectability of Western Pacific crown-of-thorns starfish (*Acanthaster* cf. *solaris*) obtained using different sampling methods (*K* = number of successive surveys on same transects).

Location	Detectability	Sampling Unit	Method for Inferring True Sample Population (*N*)	Factors Affecting Detectability
Bowden and Shell Reefs, central GBR [34]	22.7%	200 m × 10 m manta tow	Intensive, repeated SCUBA surveys	Exposure of CoTS, habitat complexity, water visibility, and reef aspect
Bowden and Shell Reefs, central GBR [34]	88.2%	20 m × 10 m SCUBA surveys	Successive SCUBA surveys (*K* = 2) recording unmarked CoTS	
Undine and Rudder Reefs, northern GBR [35]	82.0%	50 m × 5 m SCUBA surveys	Mark–recapture surveys (*K* = 6) including nighttime surveys	Size of CoTS; no effect of coral cover
Moorea, French Polynesia [47]	78.7%	50 m × 4 m SCUBA surveys	Nighttime surveys	No effect of CoTS density or habitat complexity
Wheeler Reef, central GBR [32]	81.8%	60 min timed swims	Nighttime surveys	Size of CoTS; no effect of month/season
Northern GBR [30]	50.5%	>800 m × 5 m SALAD surveys	Inferred densities based on feeding scars	Size-based differences in feeding and exposure during the day; no effect of coral cover
Lizard Island, northern GBR ^1^	78.4%	50 m × 4 m SCUBA surveys	Re-sampling (*K* = 3) following CoTS removal	Zone (depth); no effect of reef aspect or CoTS density
Rib Reef, central GBR ^1^	75.9%	250–500 m^2^ SCUBA surveys	Mark–recapture surveys (*K* = 3) including nighttime surveys	Day versus night and reef aspect; no effect of zone or coral cover

^1^ This study.

## Data Availability

The raw data will be made available by the authors upon request.

## Abbreviations

The following abbreviations are used in this manuscript:

CoTS	Crown-of-Thorns Starfish
GBR	Great Barrier Reef
MC/R	Mark Capture Re-capture
SE	Standard Error

## Appendix A

**Table A1:** Summary statistics for Lizard Island, showing the mean (±SE) density (CoTS·ha^−1^) and body size (diameter, cm) of all CoTS removed during successive surveys at each combination of site and zone (*n* = 5200 m^2^ transects). Detectability is calculated based on the proportion of the total sample population that was sighted and removed during the initial intensive survey.

Site (Aspect)	Zone	Density	Body Size	Detectability
Casuarina (leeward)	Crest	160 ± 29.15	35.25 ± 2.26	0.89 ± 0.07
	Slope	360 ± 33.7	29.43 ± 2.75	0.72 ± 0.07
Corner Beach (leeward)	Crest	170 ± 20.00	34.46 ± 1.47	0.90 ± 0.06
	Slope	200 ± 44.72	32.14 ± 1.36	0.50 ± 0.00
Lizard Head (windward)	Crest	50 ± 27.39	34.20 ± 2.39	0.89 ± 0.09
	Slope	250 ± 41.83	38.17 ± 1.97	0.70 ± 0.14
Coconut Beach (windward)	Crest	10 ± 10.00	34.20 ± 2.39	1.00 ± 0.00
	Slope	120 ± 25.50	39.42 ± 2.00	0.88 ± 0.07

**Table A2:** Summary statistics for Rib Reef, showing maximum estimates of the sample population (*N*) and corresponding estimates of CoTS density (CoTS·ha^−1^). Also shown is the mean (±SE) size of CoTS (diameter, cm) and coral cover recorded on each transect. Detectability is calculated based on the proportion of the total sample population that was recorded during initial surveys conducted during the day and at night.

Transect (Aspect)	Zone	Max *N*	Density	Body Size	Coral Cover (%)	Detectability	
						Day	Night
A-1 (Windward)	Slope	70	1400	29.73 ± 0.94	39	0.71	1.00
A-2 (Windward)	Crest	28	1120	27.33 ± 0.85	51	1.00	0.95
B-1 (Windward)	Slope	89	1780	33.67 ± 0.93	42	0.93	0.83
B-2 (Windward)	Crest	89	3560	31.37 ± 0.52	74	0.82	1.00
C-1 (Leeward)	Slope	32	640	30.33 ± 1.29	34	0.60	0.50
C-2 (Leeward)	Crest	6	240	25.83 ± 2.99	44	0.83	1.00
C-3 (Leeward)	Slope	0	0		55		
C-4 (Leeward)	Crest	4	160	28.67 ± 2.33	48	0.50	0.50
D-1 (Leeward)	Crest	54	2160	28.24 ± 1.58	51	0.60	0.28
D-2 (Leeward)	Slope	19	380	29.94 ± 1.97	21	0.77	1.00
E-1 (Leeward)	Crest	6	240	32.50 ± 2.50	44	0.50	1.00
E-2 (Leeward)	Slope	14	280	31.25 ± 1.75	16	0.36	1.00
Total		411	913.33	30.76 ± 0.39	43.26 ± 4.41	0.69	0.81
